# Capsule Endoscopy: Pitfalls and Approaches to Overcome

**DOI:** 10.3390/diagnostics11101765

**Published:** 2021-09-25

**Authors:** Seung Han Kim, Hoon Jai Chun

**Affiliations:** Division of Gastroenterology and Hepatology, Department of Internal Medicine, Korea University College of Medicine, Seoul 02841, Korea; kimseunghan09@gmail.com

**Keywords:** capsule endoscopy, magnetic assisted capsule endoscopy, locomotion, diagnostic yield, completion rate, retention, interpretation

## Abstract

Capsule endoscopy of the gastrointestinal tract is an innovative technology that serves to replace conventional endoscopy. Wireless capsule endoscopy, which is mainly used for small bowel examination, has recently been used to examine the entire gastrointestinal tract. This method is promising for its usefulness and development potential and enhances convenience by reducing the side effects and discomfort that may occur during conventional endoscopy. However, capsule endoscopy has fundamental limitations, including passive movement via bowel peristalsis and space restriction. This article reviews the current scientific aspects of capsule endoscopy and discusses the pitfalls and approaches to overcome its limitations. This review includes the latest research results on the role and potential of capsule endoscopy as a non-invasive diagnostic and therapeutic device.

## 1. Introduction

Since capsule endoscopy was introduced in the early 2000s, capsule endoscopy has played an important role in evaluating small intestinal lesions [1]. Capsule endoscopy is recommended as the first-line test for obscure gastrointestinal bleeding. It is effectively used as a diagnostic tool for small bowel diseases such as Crohn’s disease, small bowel tumor, celiac disease, unexplained abdominal pain, and diarrhea [2,3,4].

Compared to conventional endoscopy, capsule endoscopy is a less invasive examination method that does not require sedation during the examination process and reduces the patient’s discomfort. In addition, it enables easy access to structures such as the small intestine that were previously difficult to access.

Capsule endoscopy has continuously improved since it has been applied in clinical practice [5,6,7,8,9,10]. However, there are still shortcomings that need to be addressed. (1) First, the capsule endoscope cannot be positioned as intended by the examiner. (2) Unlike conventional endoscopes, air cannot be adequately inflated, limiting sufficient observation of the gastrointestinal tract. (3) Due to the device’s limitation in the form of a small pill, effective optical technology cannot be easily applied. (4) The quality of the examination is determined by the condition of the intestinal tract, such as poor bowel preparation or air bubbles. (5) Procedures such as biopsy or hemostasis are not possible. (6) There is a risk of capsule retention. (7) It takes considerable time and effort to interpret after the test is performed.

## 2. Maneuverability

### 2.1. Magnetic Navigation System

One of the disadvantages of capsule endoscopy is the impossibility of operating the device; therefore, it is difficult to observe by adjusting the field of view as desired. This affects the overall miss rate of capsule endoscopy. The miss rates for small bowel tumors, vascular disease, and ulcers were 18.9%, 5.9%, and 0.5%, respectively [11]. Efforts have been made to overcome these shortcomings to reduce the overall miss rate.

The application of magnetic fields in the medical field has long been practiced. Recently, it has been applied and used in neurosurgery and the treatment of cardiac arrhythmias [12,13]. Magnetic assisted capsule endoscopy (MACE) is an examination tool that observes the gastrointestinal tract by control the location of the capsule endoscope swallowed by the patient using a magnetic field in real-time. The magnetic field generated outside the human body makes it possible to adjust a capsule endoscope equipped with a permanent magnet or a magnetizable object using translational and rotational forces [14]. To date, several MACE systems have been developed (Table 1).

#### 2.1.1. Magnetic Maneuverable Capsule

In 2010, Swain et al. conducted a study to observe the esophagus and stomach by administering MACE by modifying colon-type capsule endoscopy (Given Imaging Ltd., Yoqneam, Israel) [15] to the human body. The capsule endoscopy involved rare earth magnetic materials and operated in an external magnetic field and transmitted images. The weight of the capsule endoscope, including the magnet, was increased to 3.5–7 g. An external paddle-shaped magnet was made to control the capsule in the human body remotely, and it consisted of two rectangular plate-shaped magnets and a handle. The size of the magnet was 100 × 100 × 30 mm. The capsule was manipulated in the esophagus for 10 min, and movement or rotation was easy. In the stomach, moving, stopping, and rotating the capsule from the pylorus to the esophageal–gastric junction was possible at any site and did not cause any discomfort to the patient.

#### 2.1.2. Magnetically Guided Capsule Endoscopy

In 2010, Siemens Healthcare and Olympus Medical Corp modified the capsule endoscope and developed a 31 × 11 mm MACE with a magnet inside [16]. The magnetically guided capsule is steered by the steering system’s dynamic magnetic field and gradient. The control system has a shape similar to that of a magnetic resonance imaging scanner, and its approximate size is 1 × 2 m. It creates a magnetic field force of approximately 100 mT (milliteslas), and in reality, approximately 3–10 mT is used to control the capsule.

#### 2.1.3. Magnetically Controlled Capsule Endoscopy System

**Figure 1:**
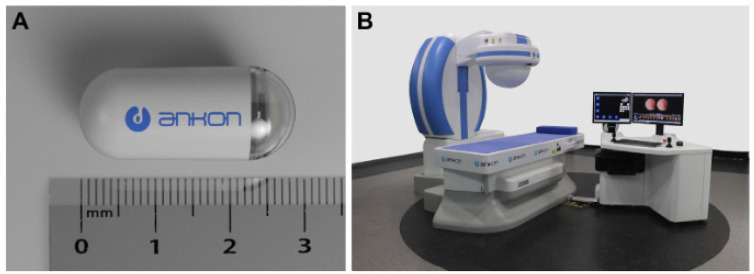
The magnetic-controlled capsule endoscopy system. (**A**) The NaviCam capsule endo-scope (Ankon Technologies Co, Ltd., Wuhan, China); (**B**) the NaviCam magnetic control system. Adapted from Liao et al. [17] with permission from Elsevier.

This system consists of a capsule endoscope, a magnet-controllable robot, a data storage device, and a computer workstation capable of real-time observation and steering (Figure 1) [17]. The size of the capsule is 28 × 12 mm, and a permanent magnet is built-in. The robotic system allows two rotational and three translational control degrees of freedom. The capsule endoscope takes two pictures per second and transmits them to the data storage device. The robotic system is a C-arm type system with an operating range of more than 50 × 50 × 50 cm. The magnetic field generated by the system is up to 200 mT or more. Liao et al. conducted a study comparing the diagnostic accuracy of conventional gastroscopy and magnetically controlled capsule endoscopy (MCE) with 350 patients complaining of abdominal discomfort [17]. The mean time to perform MCE was 26.4 ± 5.1 min, and MCE detected gastric focal lesions in the whole stomach with sensitivity of 90.4% (95% confidence interval, CI, 84.7%–96.1%), and specificity of 94.7% (95% CI, 91.9–97.5%), an 87.9% positive predictive value (95% CI, 81.7%–94.0%), a 95.9% negative predictive value (95% CI, 93.4%–98.4%), and 93.4% accuracy (95% CI, 90.83%–96.02%).

#### 2.1.4. MiroCam Navi

**Figure 2:**
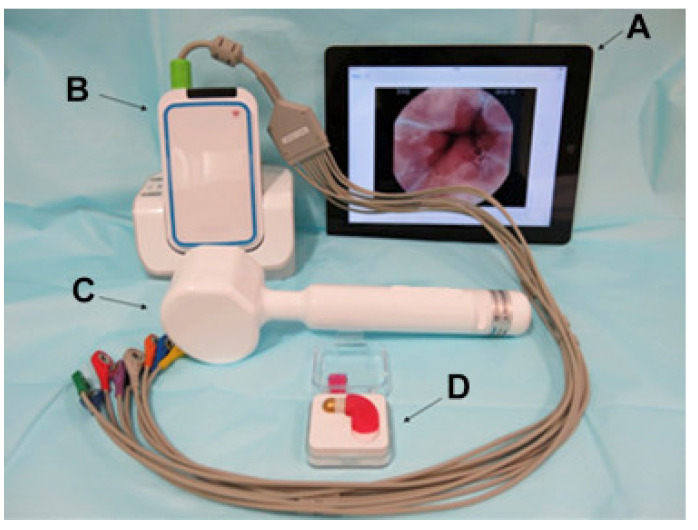
The MiroCam Navi system. (**A**) real-time viewer; (**B**) sensor and receiver; (**C**) hand-held magnet; (**D**) MiroCam Navi capsule. Adapted from Rahman et al. [19] with permission from Elsevier.

MiroCam Navi is a simple system that uses a capsule endoscope and a hand-held magnet to adjust the capsule (Figure 2). The capsule is manufactured by modifying the microcam capsule endoscope for the small intestine. Image data are transmitted to the receiver using electric field propagation, as in other microcam capsule endoscopes. The size of the MiroCam Navi capsule is 25.5 × 10.5 mm, and the weight is 4.75 g. The length of the hand-held magnet is 26 cm, the width of the handle is 3.5 cm, and the width of the head is 6.5 cm. Ching et al. compared the diagnostic yield of MiroCam Navi and gastroscopy in patients with suspected acute upper gastrointestinal bleeding [18]. A total of 33 patients were included in the study. MiroCam Navi identified more localized lesions than EGD, but the suspected lesions did not reach statistical significance. Capsule endoscopy revealed an additional cause of small bowel bleeding (18%).

### 2.2. Internal Locomotion System

Although capsule endoscopy technology has made remarkable progress, numerous unavoidable shortcomings prevent the expansion of capsule endoscopy applications. For example, capsule endoscopy cannot be observed by stopping at a certain area for diagnostic or therapeutic purposes. It is also difficult to return to an area and re-observe. These shortcomings can be overcome by adding a locomotion system to the capsule endoscope. Several research groups have tried to develop a different type of locomotion system for capsule endoscopy. However, it is still in the experimental laboratory stage because of the complexity of the gastrointestinal tract and limited power [20,21,22,23,24]. The actuator is important for propelling the capsule endoscope for an active capsule endoscope system. There are three types of internal locomotion methods.

#### 2.2.1. Inchworm-Like Capsule Endoscope

**Figure 3:**
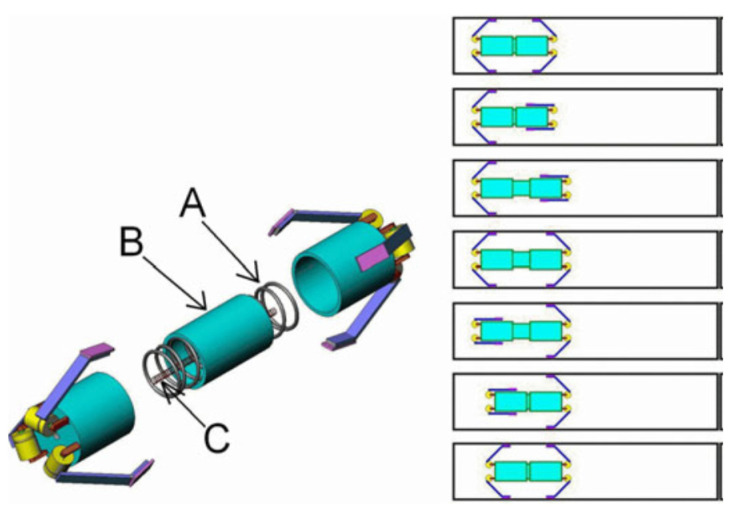
Illustration of inchworm-like locomotion mechanism. (**A**) compression spring; (**B**) capsule body; (**C**) SMA wire. Adapted from Liu et al. [25] with permission from IEEE.

Researchers prefer the friction-based locomotion method because it is based on a simple principle. The inchworm-like method works through three basic movements: anchoring, elongating, and contraction [25]. This action is performed using an actuator made of shape memory alloys (SMA). Cheung et al. presented a mechanism for locomotion and stopping the capsule endoscope in the digestive tract [26,27]. Inspired by the beetle, the authors used a micropatterned adhesive material of polydimethylsiloxane to generate an attraction force between the intestine and the capsule endoscope (Figure 3). The capsule with an inchworm-like mechanism can move back and forth by contracting and elongating the capsule body by sequentially cooling and actuating the SMA wire.

#### 2.2.2. Paddle/Legged-Based Capsule Endoscope

**Figure 4:**
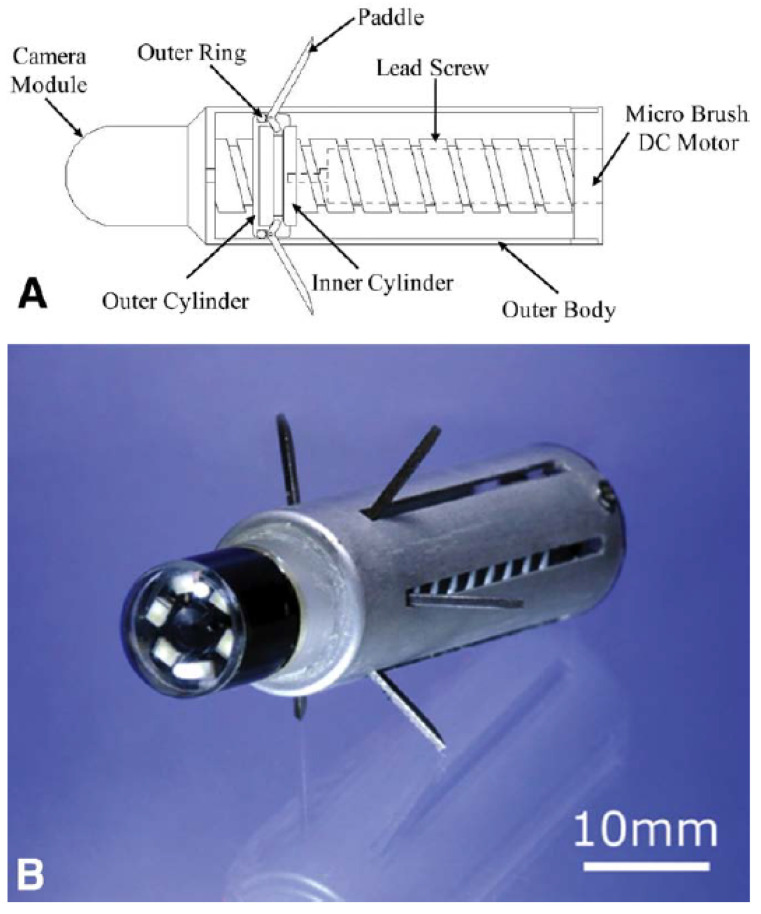
Paddling-based locomotive capsule endoscope. (**A**) Illustration of the paddling-based locomotive capsule endoscope; (**B**) the complete paddling-based locomotive capsule endoscope with fully stretched legs. Adapted from Kim et al. [22] with permission from Elsevier.

Another commonly used friction force-based locomotion method is paddle-based motion. This type of locomotion mechanism originates from canoe paddling. Several paddles or legs are included in the capsule endoscope body, controlled by the actuator and pushed backward on the gastrointestinal tract wall so that the capsule endoscope moves forward. Kim et al. presented a paddling-based capsule endoscope and tested the locomotion of active capsule endoscopy in vitro and in vivo (Figure 4) [22,24]. The locomotive capsule endoscope can easily move forward in the digestive tract by repeating this paddling operation. Therefore, the paddle connected to the outer cylinder protrudes and folds according to the direction in which the inner and outer cylinders are linearly operated along the lead screw. A positional delay from the outer cylinder to the inner cylinder occurs during linear motion owing to the gap between the inner and outer cylinders. As a result, when multiple grooves in the inner cylinder push the end of the paddle relative to the right or left, the paddle rotates at the pivot point for protrusion or folding.

#### 2.2.3. Hydrodynamic Force-Based Capsule Endoscope

**Figure 5:**
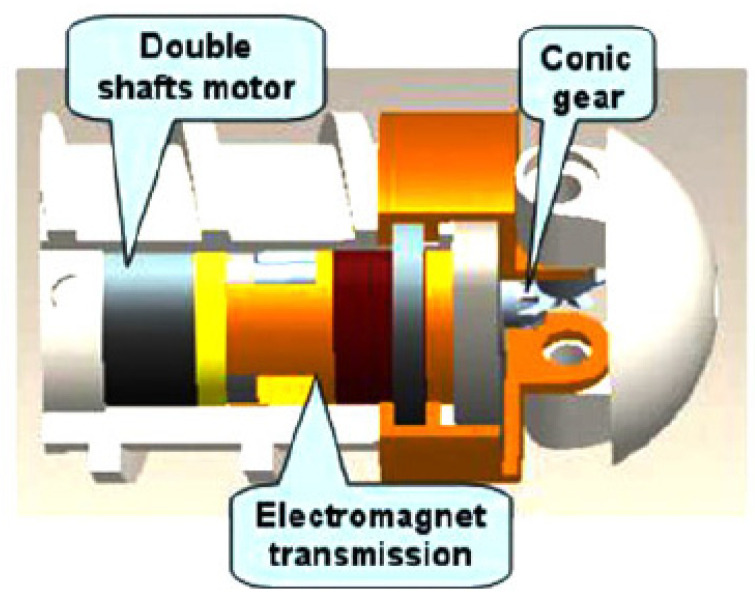
Swimming robotic capsule endoscopy. Adapted from Liu et al. [25] with permission from IEEE.

Hydrodynamic force-based systems are widely used in the design and manufacture of swimming robots. Therefore, several researchers have used this simple mechanism for the locomotion of the capsule endoscope. Chen et al. worked on a swimming robot capsule endoscope consisting of a steering head and rotational body (Figure 5) [25], divided into a steering mode and a linear mode. A micro motor is used for linear motion, and when the motor rotates, the spiral body rotates, and the capsule moves in a straight line. If the motor is reversed, the capsule endoscope is moved backward. The steering part is activated by the same motor.

## 3. Air Insufflation

**Figure 6:**
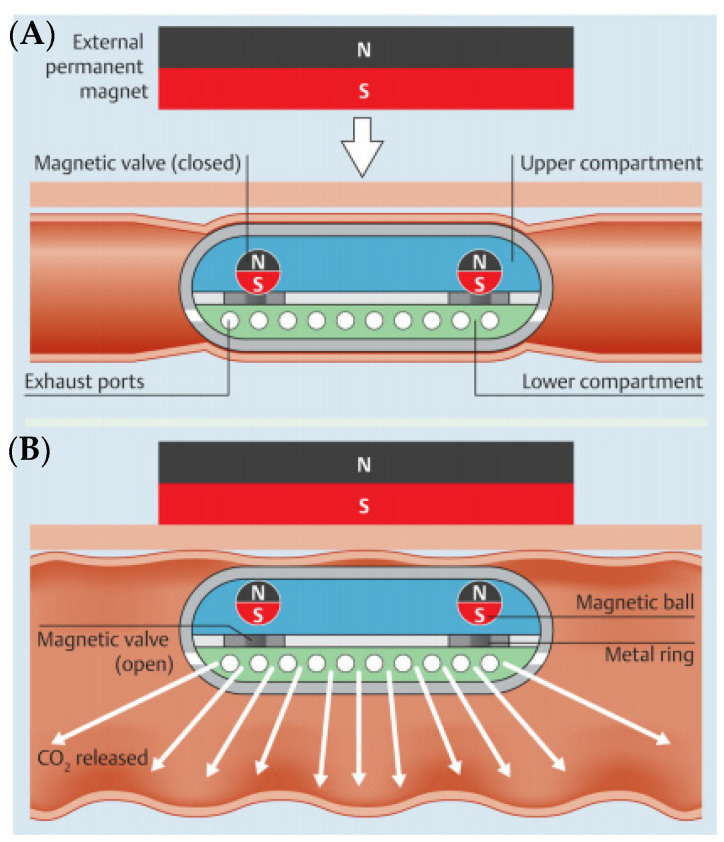
The insufflation capsule with two sections. (**A**) magnetic valves make a tight barrier between the compartments; (**B**) when activated with a permanent external magnet, the valves open to permit a mixture of the two reactants, releasing carbon dioxide. Adapted from Pasricha et al. [30] with permission from Thieme.

During capsule endoscopy, the intestinal tract is in a collapsed state, and it is difficult to observe the entire intestinal mucosa through capsule endoscopy. In a conventional endoscope, it is possible to observe the intestinal tract by injecting air to some extent, but it is impossible to inject air with a capsule endoscope [28]. Gorlewicz et al. developed a wireless insufflation capsule and performed ex vivo and in vivo animal experiments [29]. The authors provided a means of injecting air with a wireless capsule platform, using biocompatible effervescent chemistry to change the liquid and powder contained in the capsule into gas. They performed experimental evaluation of the amount of gas required to enhance the visualization and movement of the capsule and determined the amount of gas produced from a particular amount of reactant. Pasricha et al. developed a new wirelessly controlled CO_2_ insufflation system for use in colorectal capsule endoscopy (Figure 6) [30]. The inflatable capsule has two separate compartments connected by a magnetic valve. When triggered, the citric acid solution in the upper part reacts with the sodium bicarbonate in the lower part to generate carbon dioxide. It is released through the exhaust port in the center of the capsule. A permanent external magnet controls this action.

## 4. Visibility for the Diagnostic Ability

### 4.1. Upgrade of a Capsule Endoscope Device

The capsule endoscope system consists of a capsule for imaging, a device for receiving and storing data, a computer, and software. The pill-shaped capsule consists of a light source, a lens, a battery, and a transmission device. Capsule endoscopy can be used to observe the esophagus, stomach, small intestine, and large intestine. The capsule endoscope consists of (1) an optical dome, (2) lens, (3) light source, (4) image sensor, (5) battery, (6) transmitter, and (7) antenna.

The optical dome is made of hemispherical plastic in front of the capsule endoscope. There is an appropriate distance between the dome and the internal camera to observe the digestive tract with the mounted camera. The lens uses a single-focal lens with a small aperture. Light-emitting diodes are used as light sources to illuminate the inside of the digestive tract. Metal oxide silicon imagers use micropower to operate in low light and have many circuits integrated into a small chip.

The first capsule endoscopy system was produced by Given Imaging (Yokneam, Israel). The third-generation model PillCam SB3 (Medtronic, Washington, DC, USA) is currently used as a capsule endoscope for the small intestine. In addition, Mirocam (Intromedic, Seoul, Korea), CapsoCam (CapsoVision, Saratoga, CA, USA), EndoCapsule (Olympus, Tokyo, Japan), OMOM capsule (Jinshan Science and Technology, Chongqing, China) were used (Table 2). Various capsule endoscopes have been developed and used in clinical practice, but direct comparative studies on the diagnostic performance of capsule endoscopes developed by various companies have not been reported.

**Figure 7:**
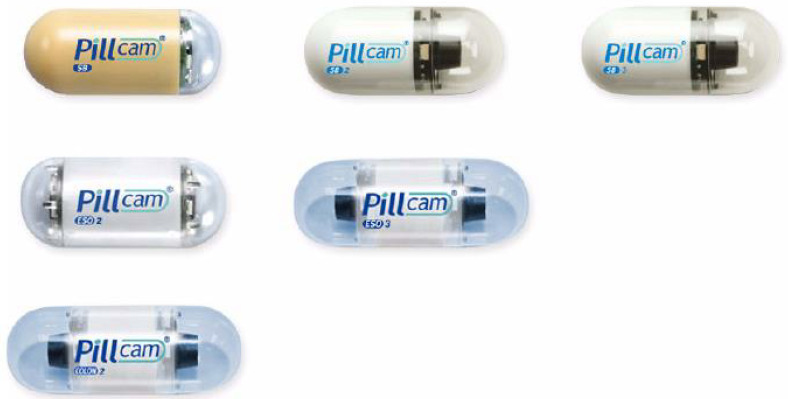
Pillcam Capsule endoscopy (image courtesy of Medtronic).

The first-generation PillCam SB capsule endoscope measures 11 × 26 mm, and both the second and third generations have the same size (Figure 7) [31,32]. The PillCam SB capsule endoscope takes two pictures per second and has a viewing angle of 140°. The second- and third-generation PillCam SB capsule endoscopes have an extended viewing angle of 156°, automatic light control, higher resolution cameras, and 12 h battery life.

Intromedic (Seoul, Korea) released the MiroCam. Other capsule endoscope models use radiofrequency transmission for image transmission, but MiroCam uses human body communication to transmit information. This technology can record videos for a longer period through finer power consumption. MiroCam measures 10.8 × 25.4 mm, has a viewing angle of 170 degrees, takes 3–6 pictures per second, and has a battery life of 12 h.

Introduced by CapsoVision (USA) in 2013, CapsoCam can store captured images in the capsule endoscope, eliminating the need to carry external receivers and storage devices during the examination period. In addition, CapsoCam provides a 360-degree panoramic view with four cameras arranged at 90° in the middle of the capsule on the side, unlike the existing capsule endoscope with a camera at the end of the capsule. CapsoCam SV-1 can take 12–20 photos per second and store the images in the capsule itself. However, there is a disadvantage in that it is necessary to find a capsule endoscope in the patient’s stool after examination and check the image taken.

### 4.2. Non-White Light Imaging

White light imaging (WLI) is currently used as the principal technology in the clinical practice of capsule endoscopy. Despite recent advances in endoscopic imaging technology, capsule endoscopy has limitations in using these technological improvements. The clinical usefulness of WLI capsule endoscopy has motivated research groups to improve integrated diagnostic capability with other sensing methods. These advancements have made it possible to overcome the drawbacks of WLI capsule endoscopy through enhanced detection of gastrointestinal pathologic lesions, such as microlesions, subepithelial lesions, and transmural pathology.

**Figure 8:**
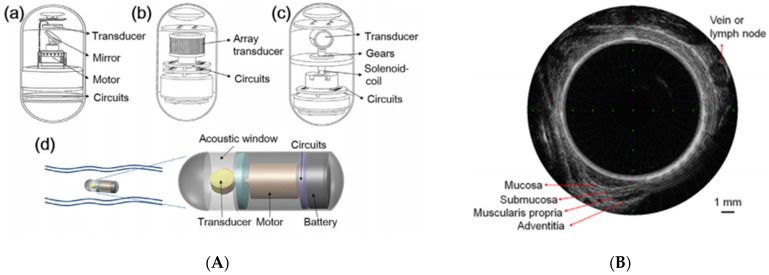
Illustration of ultrasound capsule. (**A**) (**a**) A mirror for ultrasound reflection. (**b**) Circular array transducer-based system. (**c**) A solenoid coil for rotating the transducer. (**d**) A micro-motor for rotating the transducer for circular imaging; (**B**) cross-sectional ultrasound image of a porcine esophagus. Adapted from Qui et al. [41] with permission from Elsevier.

To overcome the limitations of WLI, alternative imaging technologies, such as chromoendoscopy and narrow-band imaging (NBI), have been developed [33,34]. With NBI, mucosal surface patterns and superficial capillaries were observed more clearly, and blood vessels appeared black with increasing hemoglobin absorption [35]. NBI is widely used in clinical practice and actively applied to wireless capsule endoscopy in laboratory device manufacturing research [36,37]. With the routine application of endoscopic ultrasonography, the application of ultrasound to the capsule endoscope has been considered a necessary step to improve the diagnostic ability of the capsule endoscope beyond the optical image. Ultrasound capsule endoscopy (USCE) is still in its infancy, but several research teams are developing it [38,39,40]. Qiu et al. investigated a mechanical method using a single-element transducer powered by a vibration motor that enables radial imaging of the bowel wall (Figure 8) [41]. The frequency of the transducer plays an important role in USCE, affecting penetration depth and image resolution.

Research to integrate autofluorescence imaging into capsule endoscopy has been conducted by developing several prototype imaging devices [42,43,44], and research on applying volumetric imaging technology to capsule endoscopy using optical coherence tomography is also being conducted [45,46]. In addition, in terms of biophysical measurements, studies are being conducted to measure temperature [47,48], motility [49,50,51], slow wave [52], and pH [53,54] using capsule endoscopy.

### 4.3. 3D Reconstruction

One of the major drawbacks of capsule endoscopy is that it is difficult to accurately measure the lesion’s size. In addition, since capsule endoscopy is difficult to manipulate and inflate air, it is problematic to identify polyps or excavated lesions. To solve this problem, 3D reconstruction of capsule endoscope images has been attempted to obtain more information using current capsule endoscope images [55,56,57,58]. Koulaouzidis et al. presented a feasibility study of 3D representation software for image enhancement. They used a single image to investigate the accuracy of the software-based 3D reconstruction and the potential for 3D reconstruction to enhance the visualization of CE lesions. Three-dimensional reconstruction is desirable for CE image reviews.

Software methods, including shape-form-shading, have been used to reconstruct the 3D structure of the small bowel [59,60]. The software approach has the basic limitation of estimating accurate and powerful 3D information without adding new image information. Although this tool has shown how useful it is in analyzing capsule images, it has limited clinical significance owing to its fundamental limitations.

**Figure 9:**
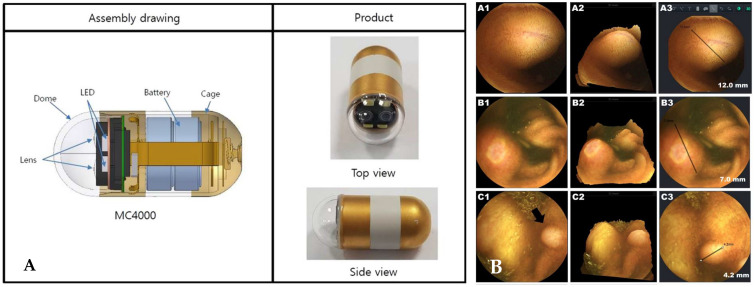
Stereo camera-based capsule endoscopy. (**A**) Illustration of MC4000 (IntroMedic^®^ Co., Seoul, Korea); (**B**) images detected by MC4000. A1, B1, and C1 present conventional capsule images (A1: subepithelial lesion, B1: ulcer of Crohn’s disease, and C1: polypoid lesion). A2, B2, and C2 show the 3D reconstructions of the conventional images. A3, B3, and C3 present an estimation of the lesion size. Adapted from Nam et al. [61] with permission from Springer Nature.

Nam et al. presented a new stereo camera-based capsule endoscope (Figure 9) [61]. The device consisted of two cameras displaced by approximately 4 mm and four LED lights. The weight and size were comparable to those of commercially available MiroCam^®^ capsule endoscopes. The census-based Hamming distance and absolute difference of intensities were used for stereo matching [62]. The authors evaluated the functioning and safety of the new capsule. In addition, the usefulness of 3D rendering and size measurement functions was assessed in each patient’s clinical setting.

## 5. Bowel Preparation

During capsule endoscopy, the diagnostic yield is reduced when food remains or air bubbles are present in the small intestine. Therefore, it is important to perform proper bowel preparation and reduce bubbles in the intestine before performing capsule endoscopy [63]. Studies have been performed to increase the completion rate, small bowel visualization quality, and diagnostic yield of CE [64,65]. Although there are conflicting results of bowel preparation studies on capsule endoscopy, it has been reported that bowel preparation using PEG effectively improves the resolution and diagnostic rate of capsule endoscopy [65]. According to a meta-analysis of the literature on bowel preparation, use of a laxative containing PEG solution and sodium phosphate improved small bowel disease resolution and diagnosis rate. In bowel preparation for capsule endoscopy, when comparing 2 and 4 L, there is no difference in image resolution, diagnostic rate, or complete examination rate of the study; therefore, it is generally recommended to administer PEG 2 L.

The use of simethicone reduces intestinal bubbles and increases the resolution of capsule endoscopy. Wu et al. conducted a literature-based meta-analysis and significantly improved the resolution of the capsule endoscope in the group administered with laxatives and simethicone (OR 2.84, 95% CI 1.74–4.65, *p* = 0.00) [66]. Therefore, concurrent administration of simethicone during bowel preparation for capsule endoscopy is generally recommended [67].

In a meta-analysis of the literature analyzing the effect of administration of prokinetics during capsule endoscopy on the diagnostic yield and completion rate of capsule endoscopy, metoclopramide showed definitive test in complete test rate compared to the control group (OR 2.8, 95% CI 1.35–3.21), and there was no difference in diagnosis rate [68]. Therefore, in patients with risk factors, prokinetics can be selectively used.

## 6. Abilities of Procedure

### 6.1. Biopsy

**Figure 10:**
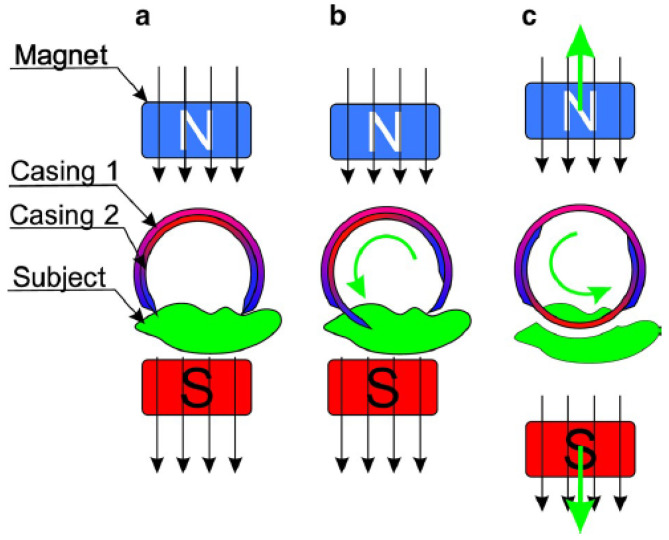
Magnetic torsion spring mechanism for a wireless biopsy capsule. (**a**) Magnetic field applied to bring the device closer to the lesion; (**b**) biopsy process; (**c**) storage of biopsy material. Adapted from Koprowski et al. [71] with permission from BMC.

Another disadvantage of capsule endoscopy is that it is impossible to perform a procedure for diagnosis and treatment that can be performed with a conventional endoscope, such as a biopsy. Several issues need to be addressed for biopsy using capsule endoscopy: (1) approaching the lesion for biopsy, (2) inserting a knife or needle for biopsy of the lesion, (3) performing biopsy, and (4) retrieval of a biopsy specimen. Simi et al. presented a magnetic torsion spring mechanism using a cutting tool [69]. This device performed a histological examination using two circular cutting knives (Figure 10). In the absence of an external magnetic field, the cutting chamber was closed. When an external magnetic field was applied, the ring-shaped knife was aligned in the direction of the external magnetic field, and the chamber opened. After removing the external magnetic field, the chamber was closed, and the tissue material was obtained and placed in the chamber. Kong et al. presented a rotational micro-biopsy device using a rotational tissue-cutting razor with a strained spring [70]. This device works by releasing the strained spring near the lesion to obtain tissue and storing it in the chamber.

### 6.2. Hemostasis

**Figure 11:**
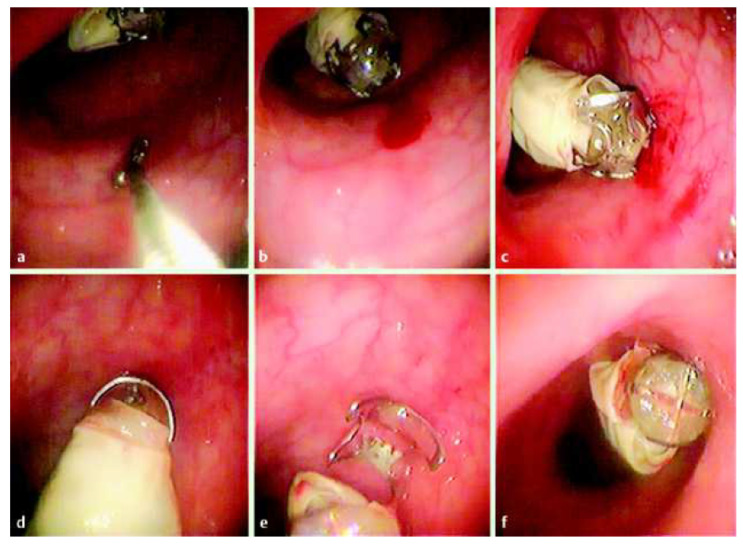
Wireless hemostatic endoscopic capsule. (**a**) Induced bleeding using a biopsy catheter; (**b**) the capsule approaching the bleeding lesion; (**c**) position adjustment using magnetic force; (**d**) capsule before the release of the clip; (**e**) deployment of the clip; (**f**) capsule without the clip. Adapted from Valdastri et al. [72] with permission from Thieme.

Valdastri et al. developed a novel wireless device that can perform hemostasis at a particular position using external adjustment by attaching a surgical clip (over−the−scope clip) (Figure 11) [72]. A surgical clip was attached to the distal end of the capsule endoscope, and it was made of nitinol, a biocompatible superelastic SMA. Ex vivo tests were repeated to test all the functions of the therapeutic capsule in terms of clip release efficiency and remote control reliability.

Leung et al. proposed an inflatable prototype capsule for hemostasis of the gastrointestinal tract via the balloon tamponade effect [73]. The device was composed of three segments connected by flexible joints. The capsule diameter was 14 mm and was surrounded by silicone rubber balloons. It inflates around hemorrhagic lesions and achieves hemostasis via the tamponade effect. Balloon inflation consists of generating gas by injecting acid into a space filled with base powder.

## 7. Retention

Capsule retention is a rare but important complication of capsule endoscopy. Capsule retention is a case in which capsules cannot be ejected for 2 weeks after administration or an intestinal obstruction occurs and surgery is required. To prevent the risk of capsule retention, a patency capsule was developed [74]. Patency capsules are made of soluble and biocompatible materials. Good patency of the intestinal tract can be checked before capsule endoscopy, and helpful information can be provided before capsule endoscopy if stenosis is suspected.

## 8. Interpretation

### 8.1. Software Upgrade

Capsule endoscopy is a convenient examination method for patients, but checking more than 50,000 images is time-consuming. After completing the capsule endoscopy, the image records stored by connecting an external storage device to the computer were analyzed and read using the software program provided by the company. The image reading program tracks the location of the intestinal tract and has a function to view continuous images from multiple sides at the same time, an enlargement function, and a function to detect bleeding and vascular suspicious lesions automatically. New functions are continuously being added (Table 3). The reading time varies depending on the reader’s experience or the type of lesion and usually takes 30 min to 2 h. Reading requires experience, and research into shortening the reading time and increasing the accuracy of diagnosis of lesions using artificial intelligence continues.

### 8.2. Artificial Intelligence

Recently, with the development of artificial intelligence, several researchers have reported the potential application of convolutional neural network (CNN) systems to diagnose various small bowel lesions. The application of this artificial intelligence capsule endoscope will reduce the time required for capsule endoscope interpretation. With the development of deep learning algorithms, the CNN, which obtains specific characteristics by polling and convolutional layers and performs back-propagation to create the best feature map, has become the main deep learning algorithm for image analysis [75,76]. Aoki et al. presented a study for finding mucosal erosion and ulcers with a CNN-based program and manually annotated more than 5000 capsule endoscopy images [77]. This program model presented promising functioning with an area under the receiver-operating characteristic curve (AUROC) of 0.958, an accuracy of 90.8%, a sensitivity of 88.2%, and a specificity of 90.9%.

Klang et al. presented a CNN model for detecting ulcers in Crohn’s disease using 17,000 capsule endoscopy images [78]. This CNN model using 5-fold cross-validation showed an AUROC of 0.99, an accuracy of 96.7%, a sensitivity of 96.8%, and a specificity of 96.6%.

Saito et al. developed a CNN model with 30,584 images to detect and classify protruding lesions [79]. This model presented an AUROC of 0.911, a sensitivity of 90.7%, and a specificity of 79.8%. For classification, sensitivities of 86.5%, 92.0%, 95.8%, 77.0%, and 94.4% were noted for polyps, nodules, epithelial tumors, submucosal tumors, and venous structures, respectively.

## 9. Conclusions

Wireless capsule endoscopy has become a routine examination in clinical practice for investigating the gastrointestinal tract. Although capsule endoscopy has several limitations, studies by several researchers to overcome these shortcomings are ongoing. For more effective technological advancement, both engineers and clinicians need to participate.

The usefulness of capsule endoscopy is emphasized because it is less invasive and is convenient during the procedure. Such usefulness will become more prominent over time.

Studies to overcome the limitations of capsule endoscopy are meaningful separately; however, they will eventually be integrated into one to bring about greater synergy. However, most of the technological advances described above are still in their infancy, and thus it is necessary to verify the efficacy of technological advances through the human application.

## Figures and Tables

**Table 1:** Comparison of magnetically guided capsule endoscopy.

	MiroCam Navi	Navicam™ Stomach Capsule System	Magnetic Maneuverable Capsule	Magnetically Guided Capsule Endoscopy
Company	Intromedic, Seoul, South Korea	Ankon Technologies, Wuhan, China	Given Imaging, Yoqneam, Israel	Siemens Healthcare, Erlangen, Germany and Olympus Medical Corp, Tokyo, Japan
Type	Hand-held magnetic field generators	Robotic magnetic capsule guidance system	Hand-held magnetic field generators	Multicoil guidance system
Human application	Yes	Yes	Yes	N/A
Year	2013	2012	2010	2010
Commercially available	Yes	Yes	N/A	N/A
FDA approval	N/A	Yes	N/A	N/A

N/A, not applicable.

**Table 2:** Currently available capsule endoscopy.

Capsule Endoscopy	PillCam SB3	MiroCam	CapsoCam SV-1	Endocapsule 10	OMOM Capsule2
Company	Medtronic	IntroMedic	CapsoVision	Olympus	Jinshan Science and Technology
Size (mm)	11 × 26	11 × 25	11 × 31	11 × 26	11 × 25
Weight (g)	3.0	3.25–4.70	3.8	3.3	4.5
Camera lens (n)	1	1	4	1	
Data transmission	Radiofrequency communication	Human body communication	N/A	Radiofrequency communication	Radiofrequency communication
Battery life (h)	11	12	15	12	10
Frame rate (frames/s)	2–6	3–6	12–20	2	2–6
Field of view (degree)	156	170	360	160	165
US FDA approval	Yes	Yes	Yes	Yes	No

FDA, Food and Drug Administration.

**Table 3:** Comparison of software specifications.

	Rapid V 8.0		MiroView 4.0	
Software mode	Suspected blood indicator	Video function for a quick review of suspected hemorrhagic lesions	Express view	A function that helps the reading by filtering out overlapping images and images of less importance among recorded images
	QuickView	Ability to play clinically important images to provide quick preview and location	SGIB	A function to help the reading of suspected bleeding lesions
	Complementary QuickView	A mode that plays back videos not provided in QuickView mode.		

## Data Availability

Not applicable.
